# The molecular mechanism of action of aspirin, curcumin and sulforaphane combinations in the chemoprevention of pancreatic cancer

**DOI:** 10.3892/or.2013.2276

**Published:** 2013-02-05

**Authors:** ARVIND THAKKAR, DHRUVITKUMAR SUTARIA, B. KARTHIK GRANDHI, JEFFREY WANG, SUNIL PRABHU

**Affiliations:** 1 Department of Pharmaceutical Sciences, College of Pharmacy, Western University of Health Sciences, Pomona, CA; 2 College of Pharmacy, The Ohio State University, Columbus, OH, USA

**Keywords:** pancreatic cancer, chemoprevention, ERK1/2, NF-κB

## Abstract

Pancreatic cancer ranks as the fourth most deadly form of cancer in the United States with ~37,000 deaths each year. The present study evaluated the chemopreventive potential of a combination of aspirin (ASP), curcumin (CUR) and sulforaphane (SFN) in low doses to human pancreatic cancer cells, MIA PaCa-2 and Panc-1. Results demonstrated that low doses of ASP (1 mM), CUR (10 μM) and SFN (5 μM) (ACS) combination reduced cell viability by ~70% (P<0.001), and also induced cell apoptosis by ~51% (P<0.001) accompanied by activation of caspase-3 and Poly(ADP-ribose) polymerase (PARP) proteins. The NF-κB DNA binding activity was inhibited by ~45% (P<0.01) and ~75% (P<0.001) in MIA PaCa-2 and Panc-1 cells, respectively. Mechanistic studies revealed that ACS promoted increase expression of phospho extracellular signal-regulated kinase 1/2 (P-ERK1/2), c-Jun, p38 MAPK and p53 proteins. Furthermore, the cells pretreated with U0126 (ERK1/2 inhibitor) partially abolished the effect of ACS on cell viability. Data from this study demonstrate that a low-dose ACS combination inhibits cell growth by inducing cell apoptosis, and proposes sustained activation of the ERK1/2 signaling pathway as one of the possible mechanisms.

## Introduction

Pancreatic cancer remains a fatal disease with a 5-year survival rate <5% (1). Chemotherapy regimens often fail to improve the outcome of pancreatic cancer patients. Only 20% of pancreatic cancer patients are eligible for surgical resection, which currently remains the only potentially curative therapy (2). The low survival rate of patients with pancreatic cancer points toward an increased need for novel therapeutics, early detection and chemoprevention strategies.

Aspirin (ASP), the traditional non-steroidal anti-inflammatory drug (NSAID), has emerged as a viable chemopreventive agent against various types of cancer (3). ASP is reported to be capable of suppressing pancreatic cancers growth *in vitro* and *in vivo*(4). Clinical studies associated with the use of ASP for pancreatic cancer chemoprevention have met with mixed results thus far (5). Given these conflicting reports on the use of ASP in pancreatic cancer but, simultaneously realizing the proven benefits as a chemopreventive agent in cancer, it reaffirms the need for further study of this drug in pancreatic cancer. Curcumin (CUR) is a diferuloylmethane derived from turmeric *(Curcuma longa)* and a pharmacologically safe agent. CUR has recently received considerable attention due to its pronounced anti-inflammatory, anti-oxidative and anti-carcinogenic activities (6,7). Sulforaphane (SFN) is a naturally occurring isothiocyanate, which is unique to cruciferous vegetables such as broccoli, cauliflower and cabbage (8). The ultimate chemopreventive effects of SFN involve multiple mechanisms, include apoptosis-inducing properties (9) and induction of cell cycle arrest.

Nuclear factor-κB (NF-κB) controls different biological processes, such as inflammation, cell cycle and apoptosis, and is a key antiapoptotic transcription factor in pancreatic ductal adenocarcinoma (10). NF-κB activation has been reported in pancreatic cancer cells, animal models of pancreatic cancer, and in human pancreatic tissue. It has been reported that mitogen-activated protein kinases (MAPK) participate in diverse cellular functions such as cell proliferation, cell differentiation and cell death (11). There are three major MAPK family subgroups: extracellular signal-regulated kinase 1/2 (ERK1/2), c-Jun N-terminal of stress-activated protein kinases 1/2 (JNK1/2) and the p38 protein kinases. Previous studies have demonstrated dual role of ERK1/2. The transient activation of ERK1/2 plays a pivotal role in cell proliferation and that sustained ERK1/2 activation induces cell cycle arrest and differentiation (12,13).

Recent literature has demonstrated that rather than administering single agents, there is increasing interest in the use of combinations of chemopreventive agents. This approach has provided means of obtaining increased efficacy by targeting multiple signaling pathways and also minimized toxicity (13,14). However, combination therapy studies specifically directed towards pancreatic cancer prevention are still in its infancy.

To date, no group has investigated the combined effects of low dose of aspirin (ASP), curcumin (CUR) and sulforaphane (SFN) on pancreatic cancer. Thus, the objectives of our study were to examine the molecular mechanism of combined effect of low doses of ASP, CUR and SFN (ACS) in the induction of apoptosis and anti-proliferative effects in MIA PaCa-2 and Panc-1 cells.

## Materials and methods

### Cell lines and cell culture

Human pancreatic cancer cell lines MIA PaCa-2 and Panc-1 were obtained from American Type Culture Collection (Manassas, VA, USA). Cells were cultured in Dulbecco’s modified Eagle’s medium (DMEM) supplemented with 10% fetal bovine serum (FBS), 1% penicillin-streptomycin at 37°C in a 5% CO_2_ humidified environment.

### Reagents and antibodies

ASP, CUR and SFN were purchased from LKT Laboratories (St. Paul, MN, USA). The chemical inhibitor U0126 was purchased from Cell Signaling Technology (Beverly, MA). Antibodies against Phospho-ERK1/2 (Thr202/Tyr204), total ERK1/2, P-p38 MAPK (Thr180/Tyr182), P-Akt (Ser474), total AKT, P-c-jun (Ser73), P-p53 (Ser15), cleaved caspase-3 (Asp175), cleaved PARP (Asp214), P-IκBα (Ser32/36) and β-actin were purchased from Cell Signaling Technology.

### Cell viability assay

The cell viability assay was performed according to the manual included with the Promega CellTitre 96 Aqueous MTS reagent (Madison, WI, USA). Briefly, 5×10^3^ cells were seeded in 96-well plates. Test compounds ASP, CUR and SFN alone and in combination ACS were added for a period of 72 h. On the last day of the incubation period, 20% MTS and 1% of phenazine methosulfate (PMS) were added to culture medium and incubated for 3 h at 37°C and measured at 490 nm. All the assays were performed in triplicates.

### Cell colony formation assay

The 1×10^4^ cells were seeded into the six-well plates in triplicate per data point. After 24 h of seeding, cells were treated with ASP, CUR and SFN alone and in combinations ACS. Two weeks after treatment, cells were fixed and stained with 0.5% crystal violet (Sigma) in methanol for 5 min. Then, colonies consisting of 50 or more cells were counted. The percentage cell survival was calculated (plating efficiency of non-treated cultures = 1).

### Flow cytometric analysis for apoptosis

The detection was performed according to Annexin V-fluorescein isothiocyanate (FITC) Vybrant Apoptosis assay kit #3 (Invitrogen, Grand Island, NY, USA). MIA PaCa-2 and Panc-1 cells (~3×10^5^) were seeded in six-well plates and treated with ASP, CUR and SFN alone and in combination ACS for 48 h. The cell suspension of 1×10^5^ cells was then subjected to 5 μl of FITC Annexin V and 1 μl of the 100 μg/ml PI followed by incubation in the dark for 15 min. The samples were analyzed using Beckman Coulter Cytomics FC500 (Brea, CA, USA).

### NF-κB activation assay

The DNA-binding activity of NF-κB in pancreatic cancer cells was quantified by ELISA, using the TransAM NF-κB p50 transcription factor assay kit (Active Motif, Carlsbad, CA, USA). Briefly, 10 μg of total protein was incubated in 96-well plates coated with immobilized oligonucleotide for the p50 subunit. NF-κB binding to the target oligonucleotide was detected by incubation with primary antibody specific for the activated form of p50 (Active Motif), visualized and quantified at 490 nm.

### Western blot analysis

MIA PaCa-2 and Panc-1 cells were treated with ASP, CUR and SFN alone and in combination for 4, 8, 24 and 48 h. Cells were lysed in RIPA buffer and were fractionated onto SDS-PAGE gels and then transferred to nitrocellulose membranes. The membranes were blocked with 2% bovine serum albumin (BSA) in Tris-buffered saline (TBS)-Tween-20 and probed with primary antibodies (1:1000 dilution) followed by horseradish peroxidase (HRP)-labeled secondary antibodies (1:5000 dilutions). The blots were probed with the Super Signal West Pico Chemiluminescent substrate (Thermo Scientific, Pittsburgh, PA, USA) to visualize the immunoreactive bands.

### Statistical analysis

Results were expressed as mean ± SEM. A one-way ANOVA followed by Dunnett’s multiple comparison test *post hoc* analysis using Graph pad prism software (La Jolla, CA, USA) was performed to analyze and compare the results. A P-value of ≤0.05% was considered significant.

## Results

### Combination of ASP, CUR and SFN shows a synergistic effect on the reduction of cell viability

**Figure 1:**
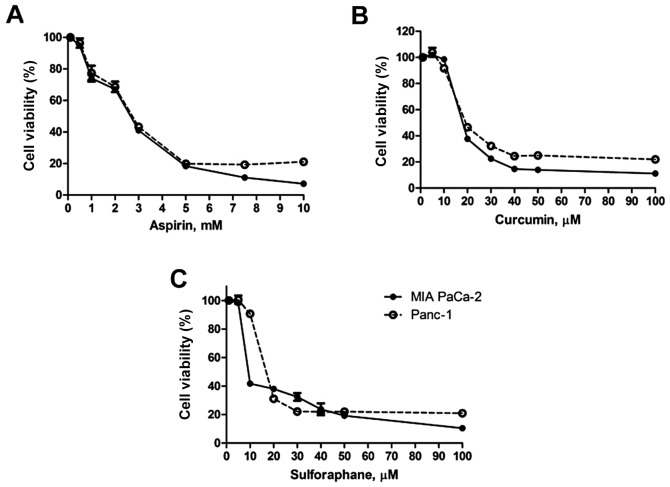
Dose-dependent inhibition of cell viability in pancreatic cancer cells. MIA PaCa-2 and Panc-1 cells were treated with (A) aspirin, (B) curcumin and (C) sulforaphane for 72 h.

In order to evaluate the effects of ASP, CUR and SFN on pancreatic cancer cells, we treated MIA PaCa-2 and Panc-1 with various concentrations of the ACS treatment for 72 h, and measured cell growth by MTS assay. A dose-dependent reduction of the growth of MIA PaCa-2 and Panc-1 cells (Fig. 1A-C) was observed. In case of MIA PaCa-2, the IC_50_ concentrations for ASP, CUR and SFN observed were 2.6 mM, 19.6 μM and 10.7 μM, respectively. Similarly, in Panc-1 cells, the IC_50_ values for ASP, CUR and SFN were calculated to be 2.4 mM, 19.6 μM and 16.0 μM, respectively. Next, to examine the effect of combined regimen on cell proliferation, MIA PaCa-2 and Panc-1 cells were treated with ineffective concentrations of ASP (1 mM), CUR (10 μM) and SFN (5 μM) for 72 h.

**Figure 2:**
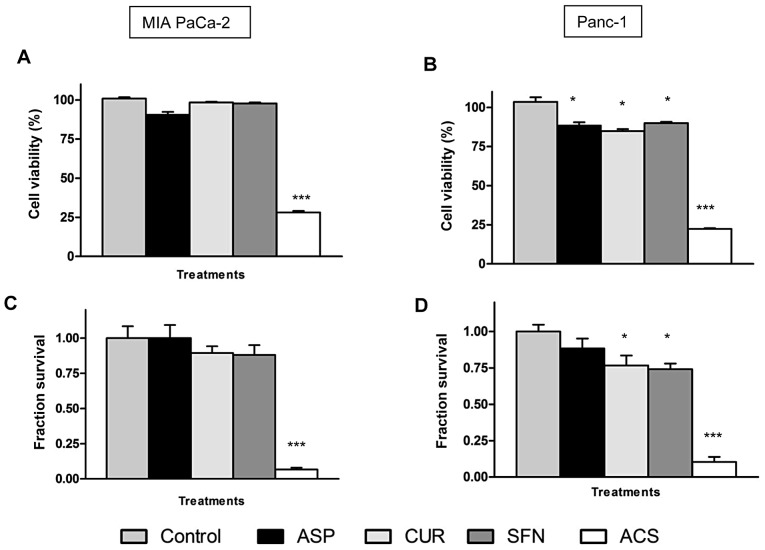
Low dose ACS combination inhibits cell viability. Cell viability of (A) MIA PaCa-2 and (B) Panc-1 cells was analyzed after treatment with aspirin (ASP), curcumin (CUR) and sulforaphane (SFN) individually and in combination of ACS (ASP+CUR+SFN) for 72 h. Each bar represents the mean percent viable cells measured in three parallel but independent experiments. Cell colony assay of (C) MIA PaCa-2 and (D) Panc-1 cells showing survival fraction of ASP, CUR and SFN individually and in combination ACS. Statistical significance was determined by one-way ANOVA followed by Dunnett’s multiple comparison test *post hoc* analysis.^*^P<0.05; ^***^P<0.001 represents statistical significance of differences between control and treatment group.

As shown in Fig. 2A, individually, the chemopreventive agents did not show significant change in cell viability at these concentrations, thereby demonstrating an ineffective profile. However, when used in combination at same concentrations, ACS showed a significant synergistic effect with a reduction in cell viability of MIA PaCa-2 cells by as much as ~70% (P<0.001). Fig. 2B demonstrated similar results with Panc-1 cells, where combinations of ACS showed a remarkable decrease of ~75% (P<0.001) in cell viability. Notably, when only dual combination studies of ASP with either CUR or SFN were conducted at the same concentration, there was no significant decrease in cell viability, with only ~20% decrease being observed from dual combinations (data not shown). Thus, the triple combination of ACS administered at low concentrations showed a significant reduction in cell viability.

To evaluate long-term efficacy of ACS on cell survival, a clonogenic assay was performed. The survival fraction of the control group was set at 1 (representing 100% cell survival) and the cell survival fraction was calculated based on individual and combination treatment. Quantitatively, an evaluation of cell survival on MIA PaCa-2 cells showed survival fractions of 0.92 (ASP), 0.89 (CUR) and 0.88 (SFN), whereas ACS combination showed significant decrease in the survival fraction of 0.06 (P<0.001) (Fig. 2C). Similar results were observed in case of Panc-1 (Fig. 2D) with low survival fractions of cells.

### ACS induces significant apoptosis in pancreatic cancer cells

**Figure 3:**
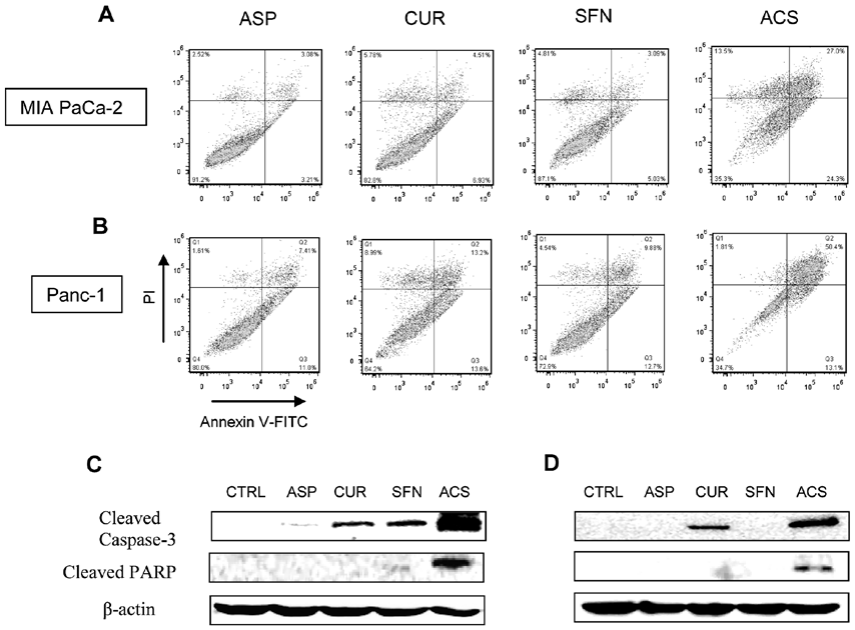
Chemopreventive ACS combination induces apoptotic cell death. (A) MIA PaCa-2 and (B) Panc-1 cells were treated with aspirin (ASP), curcumin (CUR) and sulforaphane (SFN) or in combination ACS for 48 h and stained with Annexin V-PI kit. The (C) MIA PaCa-2 and (D) Panc-1 cells showed activation of caspase 3 and PARP proteins with combination ACS. Equal loading was confirmed by using β-actin antibody.

The induction of apoptosis was measured by flow cytometry for all individual drugs and their combinations on MIA PaCa-2 and Panc-1 cells. Individual concentrations of ASP, CUR and SFN showed minimal apoptotic cells. In case of MIA PaCa-2 cells (Fig. 3A), ASP, CUR and SFN showed approximately 8, 17 and 13% cell death, respectively. However, when mixed in combinations, the ACS combinations demonstrated ~51% cell death (P<0.01). In case of Panc-1 cells (Fig. 3B), ACS combination showed ~63% of apoptotic cells (P<0.01). Overall, our studies confirmed that ACS combinations were extremely effective in inducing apoptosis of cancer cells. Since caspase activity contributes to the overall apoptotic morphology by cleavage of various cellular substrates, we examined the effect of ACS treatment on caspase-3 activation and proteolytic cleavage of PARP using western blotting. As shown in Fig. 3C and D, there were marked increase in the levels of cleaved caspase-3 and cleaved PARP in ACS treatment compared with individual drug alone.

### ACS inhibits NF-κB activity in pancreatic cancer cells

**Figure 4:**
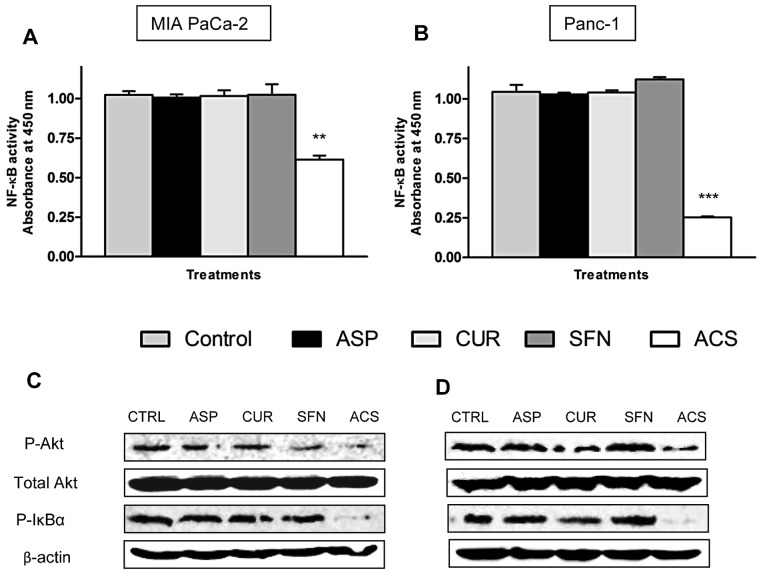
Combined effect of ACS on NF-κB signaling pathway. (A) MIA PaCa-2 and (B) Panc-1 cells were treated with aspirin (ASP), curcumin (CUR), and sulforaphane (SFN) individually or combination ACS for 48 h. Total protein was extracted and incubated in 96-well plate coated with DNA binding site for the p50 subunit of NF-κB. Data are shown as mean ± SEM (n=3). (C) MIA PaCa-2 and (D) Panc-1 blots were probed for expression of P-IκB and P-Akt by western blotting. The equal loading was confirmed by using β-actin antibody. Statistical significance was determined by one-way ANOVA followed by Dunnett’s multiple comparison test *post hoc* analysis. ^**^P<0.01; ^***^P<0.001 represents statistical significance between control and ACS treatment.

To gain further insight into the mechanism associated with the combination ACS, the DNA binding activity of p50 subunit of the NF-κB complex was evaluated. As shown in Fig. 4A, ~45% (P<0.01) decrease in the p50 binding activity was observed in MIA PaCa-2 cells in the presence of the ACS combination, whereas ~75% (P<0.001) decrease in NF-κB activity occurred in Panc-1 cells (Fig. 4B). These results were confirmed by checking levels of phosphorylated IκBα by Western blotting. As shown in Fig. 4C and D, MIA PaCa-2 cells and Panc-1 cells showed constitutive levels of P-IκBα, whereas treatment with ACS reduced the expression of P-IκBα. Moreover, Akt has been reported to be linked to the activation of IκBα and NF-κB (15). Thus, studies were conducted to examine if combination ACS inhibits phosphorylation of IκBα through inhibition of Akt activation. As presented in Fig. 4C and D, Akt was constitutively active in MIA PaCa-2 and Panc-1 cells respectively, and combination ACS inhibited phosphorylation of Akt, whereas levels of total Akt remained the same.

### ACS induces ERK1/2 activation in pancreatic cancer cells

**Figure 5:**
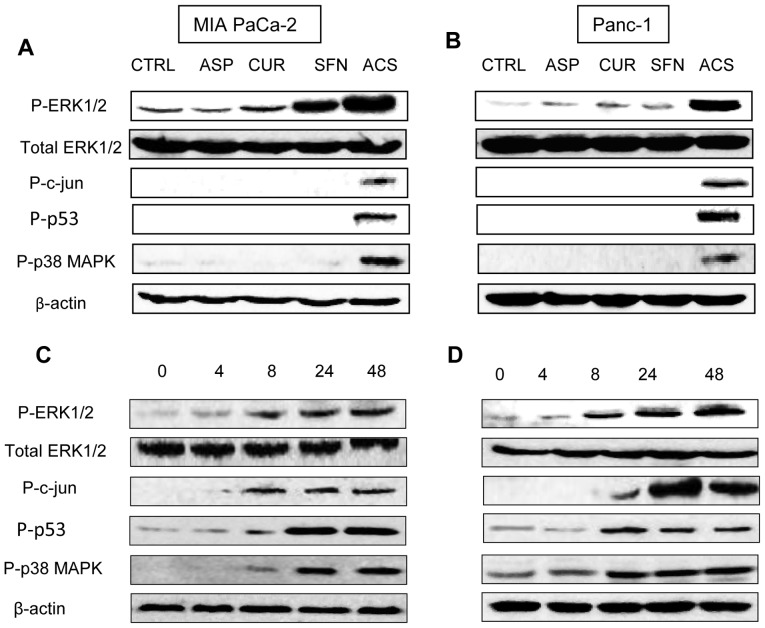
ACS combination activates ERK1/2, c-jun, p53 and p38 MAPK. (A) MIA PaCa-2 and (B) Panc-1 cells were treated with ASP, CUR and SFN alone or in combination ACS for 48 h. The expressions of P-ERK1/2, P-c-jun. P-p53 and P-p38 MAPK were increased after ACS treatment. (C) MIA PaCa-2 and (D) Panc-1 confirms the activation of P-ERK1/2, P-c-jun, P-p53 and P-p38 MAPK at various time intervals. The equal loading was confirmed by using an anti-β-actin antibody.

The ASP, CUR and SFN are reported to modulate ERK-MEK pathway (16–18), hence we wanted to determine whether the ERK-MEK pathway is involved in mediating growth inhibition by combination ACS, MIA PaCa-2 and Panc-1 protein lysates were analyzed by western blot analysis. We observed that incubation of MIA PaCa-2 and Panc-1 cells with combination ACS produced higher phosphorylation of ERK1/2 compared with the control and individual drugs (Fig. 5A and B). We also observed increased in phosphorylation of c-jun, p53 and p38 MAPK proteins. Next, we investigated the activation of ERK1/2 at different time intervals for 4, 8, 24 and 48 h. The expression of phospho-ERK1/2 was profoundly increased after 8 h of ACS treatment, and the ERK activation was persistent for 48 h after ACS treatment. Total ERK1/2 activity remained unchanged during all of these conditions (Fig. 5C and D).

### Involvement of ERK1/2 activation in pancreatic cancer cell viability

**Figure 6:**
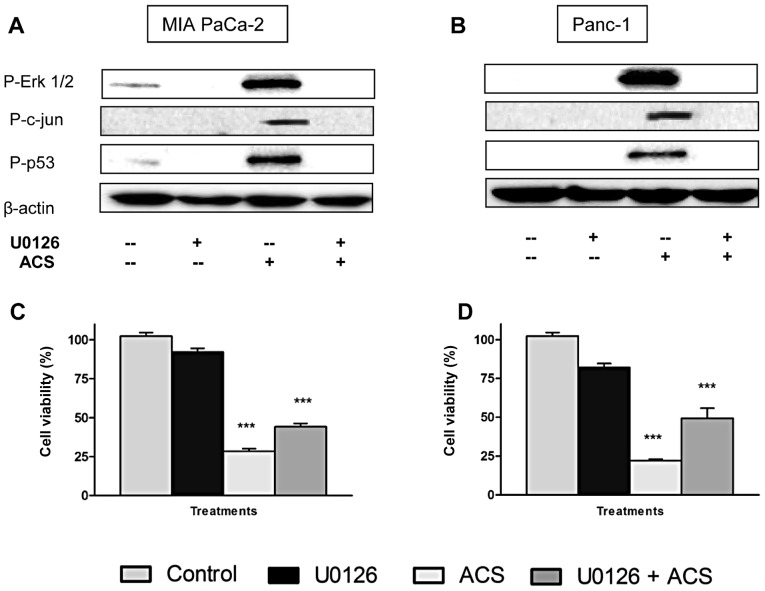
Combined effect of U0126 and ACS on cell viability. Western blot and cell viability assay of (A and C) MIA PaCa-2 and (B and D) Panc-1 were pretreated with U0126 (10 μM) for 45 min before ACS treatment. Results are expressed as the mean ± SEM from two independent experiments. The Equal loading was confirmed by using an anti-β-actin antibody. Statistical significance was determined by one-way ANOVA followed by Dunnett’s multiple comparison test *post hoc* analysis. ^***^P<0.001 represents statistical significance between control and ACS treatment

In order to verify the involvement of ERK1/2 activation in ACS-induced apoptosis, we used MEK1/2 inhibitor, U0126, and analyzed by western blot and cell viability assay. The U0126 blocks MEK1/2 phosphorylation and subsequent activation of ERK1/2. MIA PaCa-2 and Panc-1 cells were exposed to combination ACS for 48 h after pretreatment with U0126 (10 μM) for 45 min. As shown in Fig. 6A, ACS-induced phosphorylation of ERK1/2, c-jun and p53 were dramatically blocked by U0126 pretreatment in MIA PaCa-2 cells. There was no effect of U0126 on the amount of total ERK1/2 (Fig. 6A). Similar results were observed in Panc-1 cells (Fig. 6B). These studies demonstrated that ACS activation of ERK1/2 was dependent on MEK1/2.

As U0126 was able to inhibit ERK1/2 phosphorylation induced by ACS, we next investigated whether the U0126 could attenuate ACS-induced reduction in cell viability. As shown in Fig. 6C and D, U0126 alone did not alter the cell proliferation of MIA PaCa-2 and Panc-1 cells, but pretreatment with U0126 partially attenuated ACS induced reduction in cell viability.

## Discussion

Pancreatic cancer ranks the fourth in mortality from cancer in the United States with ~37,000 deaths each year. Early diagnosis of this disease is difficult because it develops without any early symptoms. Survival of patients with pancreatic cancer is <5% over 5 years which makes this disease of great concern (1). Therapeutic outcomes with pancreatic cancer are not useful for patients especially upon a late diagnosis thus strategies to prevent this disease from occurring have become an important area of research.

Our research is focused on combination treatment using ACS to study its effects against pancreatic cancer cells MIA PaCa2 and Panc-1. Low concentration of single agent has largely been demonstrated to be ineffective, hence the hypothesis that two or more agents when delivered at low concentrations together, may exhibit an additive or synergistic effect against the cancer cells. This can be attributed to the multi-factorial nature of carcinogenesis wherein cancer occurs as a result of multiple cellular changes during a prolonged time period. The cell proliferation studies demonstrated that low concentration of ASP, CUR and SFN when used alone did not reduce the cell viability, however, when combined together at same low concentration, a significant synergistic reduction in cell viability in MIA PaCa-2 and Panc-1 cells were observed (P<0.001). Many investigators have reported ASP, CUR and SFN alone to be effective against pancreatic cancer; however they have used significantly higher drug concentrations than used in this study. Concentrations of ASP (2–5 mM) and CUR (20–50 μM) have been reported in the literature to be active against pancreatic cancer cells (7,16,17,19,20). From our studies, we report a 2–5-fold reduction in doses with ASP (1 mM) and CUR (10 μM). Similarly, 20–40 μM of SFN is reported to be active against various cancers, whereas our dose of 5 μM concentration in combination with ASP and CUR reduced the concentration by 4–8 times (18,21,22). Subsequent studies using apoptosis and colony formation assays confirmed these observations and further strengthen the hypothesis of synergistic effect using combinatorial regimens.

Akt plays critical roles in mammalian cell survival signaling and has been shown to be activated in various cancers (10). Activated Akt promotes cell survival by activating the NF-κB signaling pathway (15,23) and by inhibiting apoptosis through inactivation of several pro-apoptotic factors including Bad, Forkhead transcription factors and caspase-9 (24). The Akt kinase has also been considered an attractive target for cancer prevention and treatment. Several studies also suggest that curcumin has molecular targets within the Akt signaling pathways, and the inhibition of Akt activity may facilitate inhibition of proliferation and induction of apoptosis (25–27). Bava *et al* reported that curcumin downregulated Taxol-induced phosphorylation of Akt, which interacts with NF-κB, suggesting that enhanced anti-tumor activity by curcumin is through the inactivation of P-Akt and NF-κB pathways (28). In addition, SFN has been reported to induce apoptosis in pancreatic cancer cells by inhibiting caspase-3 and P-Akt (21,29,30). In concordance with these reports, we also demonstrate that the ACS combination downregulates activity of P-Akt and NF-κB.

Our study also presents a plausible mechanism by which ACS combination can induce apoptosis in MIA PaCa-2 and Panc-1 cells, through activation of the ERK1/2 signaling system. It is important to note that there are different mechanisms of ERK activation, such as induction by growth factors could be rapid (occurring within minutes) and transient, which leads to cell proliferation and survival (31). However, persistent or sustained ERK1/2 activation that last >12 h is involved in cell differentiation and death (32). The ACS combination initiates ERK1/2 induction at 8 h, and the activity remains highly elevated through the remaining time period examined (48 h). Moreover, ERK1/2 pathway is partially responsible for ACS-induced apoptosis, as the suppression of proliferation is partially abrogated by inhibitor of the MEK/ERK pathway (U0126). This finding further emphasized the importance of ERK1/2 activation and its activity in ACS induced apoptosis of MIA PaCa-2 and Panc-1 cells. Our results support the pro-apoptotic role of ERK1/2 during ACS treatment and are in agreement with previous studies, which demonstrate that ERK activation is required for cisplatin-induced apoptosis in HeLa and A549 cells (33). Our results also add to the growing evidence about the involvement of sustained activation of ERK1/2 in regulating the apoptosis. Thus, these results provide a rationale that the low-dose ACS combination could be developed as a potential treatment against human pancreatic cancer.
